# Use of Aflibercept to Treat Retinopathy of Prematurity in Children with Extremely Low Birth Weight

**DOI:** 10.3390/jcm15051912

**Published:** 2026-03-03

**Authors:** Maria Szwajkowska, Beata Jaroszewska-Świątek, Małgorzata Woś

**Affiliations:** 1 Department of Head and Neck Surgery for Children and Adolescents, University of Warmia and Mazury, 10-561 Olsztyn, Poland; 2 Prof. Stanislaw Popowski Regional Specialized Children’s Hospital, 10-561 Olsztyn, Poland; 3 Department of Clinical Pathology and Birth Defects in Newborns and Infants, University of Warmia and Mazury, 10-561 Olsztyn, Poland; 4 Ophthalmology Department with a Children’s Unit, S. Żeromski Specialist Hospital, 31-912 Krakow, Poland

**Keywords:** retinopathy of prematurity, ROP, anti-VEGF, aflibercept

## Abstract

**Background:** Aflibercept is one of the anti-VEGF drugs used, among others, for the treatment of retinopathy of prematurity, alongside the widely used bevacizuab and ranibizumab. It is a recombinant fusion protein composed of the human VEGFR-1 and VEGFR-2 domains combined with the Fc part of human IgG, called VEGF-TRAP. The paper describes a group of premature infants treated with aflibercept due to retinopathy of prematurity at the Regional Specialized Children’s Hospital in Olsztyn, Poland, in the years 2017–2019. **Methods:** Eleven children (22 eyes) with extremely low birth weight and type 1 ROP and A-ROP qualified for treatment. The birth weight of the children was 460–940 g (average 677 g). Children were treated between 32 and 38 weeks of postconceptional age (on average in 33.3 week). We administered 1 mg (0.025 mL) of aflibercept intravitreal to each eye under local anesthesia. **Results:** In all cases, the retinopathy regressed. Between 2 and 7 weeks after treatment, the disease reactivated in five children (45%) in the form of ROP type 2, and these children underwent retinal laser photocoagulation. One child had a complication in the form of a cataract in one eye, while the remaining children had no complications after the injection and laser therapy. In all children, there was complete regression of ROP, and retinal vascularization was observed up to the end of zone III or up to the border of laser therapy. A child with cataract underwent lensectomy. All children are under the care of our center, and were examined ophthalmologically and strabologically; the results of these tests will be presented in a separate study. **Conclusions:** Aflibercept differs from other anti-VEGF drugs in its point of action and pharmacodynamics of action. In the literature, its effectiveness is estimated at over 80%, but there are also studies in which the reactivation of ROP after treatment reaches over 40%. In this study, the treated children were characterized by extremely low birth weight and, as a result, numerous complications related to prematurity occurred, such as bronchopulmonary dysplasia, sepsis, anemia, heart defects, and others. Many of them were in the intensive care unit. These factors may influence ROP reactivation and complications. The description compares studies in which aflibercept was administered at the same dose—1 mg. Currently, large studies (e.g., Firefleye and Butterfleye) describe the effects of a new aflibercept therapy at a dose of 0.4 mg. Studies on aflibercept obviously require further observations, so all reports, even from small groups, are important.

## 1. Introduction

Retinopathy of prematurity is a condition caused by immaturity of the retina in children born prematurely and involves the proliferation of retinal vessels, which may lead to retinal detachment and, consequently, to significant visual impairment and even blindness.

The vascular endothelial growth factor (VEGF) plays a role in angiogenesis in fetal life. Retinal vascularization begins in the 16th week of fetal life, and it ends in the 32–34th week on the nasal side and 38–40th week on the temporal side.

Intravitreal anti-vascular endothelial growth factor (anti-VEGF) drugs have been used in ophthalmology since 2004 (pegaptanib), and since 2005, new and improved ones have appeared, such as bevacizumab, ranibizumab, and aflibercept. These preparations are used in retinal diseases such as wet AMD, secondary macular edema retinal vessel occlusions (emboli and/or clots of the central vessels or their branches), diabetic macular edema, and choroidal neovascularization secondary to myopia. Indications for anti-VEGF preparation use in premature infants are clearly defined and include aggressive forms of ROP (A-ROP), rapid progression of lesions with the so-called “plus” disease, disease progression despite laser therapy, and the occurrence of changes in the eye (narrow pupil, rubeosis iridis, opaque optical centers), making laser therapy impossible. Treatment of ROP with anti-VEGF can be used as monotherapy or in combination with laser therapy (before or after injection).

Laser therapy of the avascular peripheral retina is still the “gold standard” of ROP treatment in Poland and worldwide. However, anti-VEGF drugs, due to their beneficial effects, are increasingly the subject of research and discussion.

Treating retinopathy of prematurity involves various anti-VEGF drugs currently used around the world, as well as new drugs being introduced (e.g., brolcizumab, conbercept) [1]. Most scientific reports concern the use of bevacizumab and ranibizumab as they have been on the market longer (bevacizumab since 2005, ranibizumab since 2006), as well as aflibercept (since 2012 worldwide, but only since 2013 in Poland).

In 2017, in our hospital, we obtained approval from the Bioethics Committee to treat retinopathy of prematurity with both aflibercept and ranibizumab, but due to the better availability of the drug at that time, patients received aflibercept (Eylea, Bayer) until 2019. On 4 August 2019, the European Commission (European Medicines Agency) approved ranibizumab as the first and only registered anti-VEGF preparation in the pharmacological treatment of ROP. From this point on, our patients with retinopathy of prematurity receive only ranibizumab if they have an indication for anti-VEGF therapy.

Currently, however, more and more countries are beginning to use aflibercept more widely in children with retinopathy of prematurity. In 2022, efforts to obtain a permit for its use in ROP began in the European Union and Japan, and in February 2023, aflibercept was approved by the FDA for treating retinopathy of prematurity in the USA.

Unlike bevacizumab (full-length antibody) and ranibizumab (fragment of an antibody (Fab)), aflibercept is a recombinant fusion protein composed of the human VEGFR-1 and VEGFR-2 domains fused to the Fc part of human IgG. After intravitreal administration, aflibercept is slowly absorbed from the vitreous into the circulatory system and is present in the systemic circulation, mainly in the form of an inactive, stable complex with VEGF, while only “free aflibercept” can bind to endogenous VEGF. Within 1 to 3 days after intravitreal injection of 2 mg of the drug (adult dose), free aflibercept plasma concentrations decrease to the limit of quantification. The elimination time from the vitreous body is approximately 7 days, and the maximum plasma concentration occurs 1–3 days after injection. Free aflibercept is detected in peripheral blood up to 4 weeks after intravitreal injection [2,3].

VEGF is known to play a role in the normal development of the kidneys, lungs, and brain. It was found that the level of anti-VEGF in the serum in premature infants after intravitreal injection had increased after a week, making it necessary to consider the drug’s effects on the development of other organs. This is why the drug should be used with caution in children and requires long-term observations of their general condition, especially the development of the nervous system.

## 2. Purpose

The aim of this study is to present cases of treatment with aflibercept in 11 children (22 eyes) born prematurely, with extremely low birth weight, suffering from various stages of retinopathy of prematurity, and staying at the Neonatal Pathology Department of the Regional Specialized Children’s Hospital in Olsztyn in the period 2017–2019. Before administering the drug, we obtained written consent from parents or legal guardians to administer the drug “off label”.

**Figure 1:**
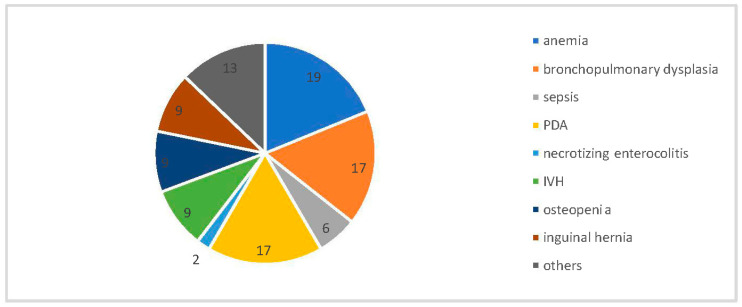
General diseases of premature infants treated with aflibercept (in percent).

All of the children were born between 23 and 27 weeks of postconceptual age (average 24.8 weeks), and their birth weight was 460 g to 940 g (average 677 g). The children were suffering from many diseases typical of premature babies with extremely low birth weight, summarized in the diagram below (Figure 1). Eight of the examined children stayed in the intensive care unit.

## 3. Method and Results

We qualified children with ROP in zone I with a plus disease, stage 3 without a plus symptom in zone I, stage 2 or 3 with a plus symptom in zone II (ROP 1), and an aggressive form of ROP appearing in ROP type 2 for treatment with aflibercept.

The children were examined ophthalmologically from approximately 4 weeks of age. Subsequent check-ups took place approximately every 5–10 days, depending on the child’s ophthalmological and/or general condition. Patients were qualified for aflibercept treatment between 32 and 38 weeks of postconceptual age, with an average PCA of 33.3 weeks. Aflibercept was administered intravitreal at a dose of 1 mg (0.025 mL) under local anesthesia and under patient sedation, if possible, after disinfecting the skin with Microdacyn (sodium hypochlorite, hypochlorous acid) and the conjunctival sac with a 5% povidone-iodine solution. In all children, the drug was administered to both eyes. The patient remained in their bed, and the injection was administered in the sterile conditions. After the injection, levofloxacin was administered four times a day for 3 days into the conjunctival sac.

All children were monitored to measure their heart rate, blood pressure, oxygen saturation, and respiration. No disturbing symptoms were observed on the patient’s part, other than during the ophthalmological examination (even smaller increases in blood pressure were observed due to the shorter procedure).

**Figure 2:**
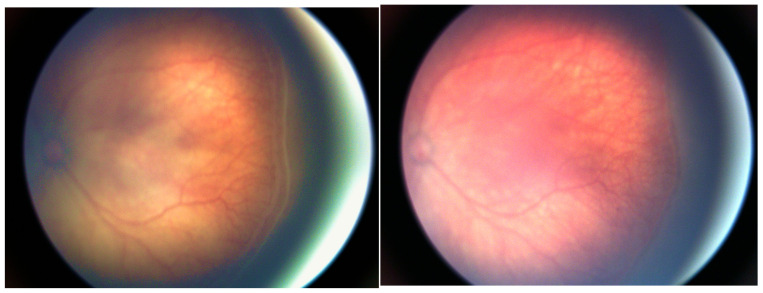
Retina before aflibercept administration and 7 days after injection.

In all children treated with aflibercept, there was regression of retinopathy of prematurity—i.e., withdrawal of the “plus” disease and growth of retinal vessels, as shown in Figure 2.

However, after a few weeks, 5 of them (45%) experienced reactivation of the disease in both eyes in the form of a demarcation line and the development of ROP 2 in zones II and III; these children underwent transpupillary laser photocoagulation. Gender, birth weight, gestational age, stage of ROP, and type and duration of treatment are summarized in Table 1.

Immediately after the injection, a small subconjunctival hematoma occurred in several cases. No local complications such as eyelid edema, conjunctival edema, subconjunctival hemorrhage, conjunctivitis, intravitreal hemorrhage, retinal hemorrhage, or retinal detachment occurred in any child. In one case (after intravitreal injection and laser therapy), a cataract developed in one eye, and the child is currently undergoing lens removal surgery. It was a patient recovering following RSV infection, with suspected metabolic disease.

None of the children experienced any deterioration of their general condition after administration of the drug. None of the children died. All of them are currently continuously checked ophthalmologically.

## 4. Discussion

Aflibercept is increasingly used to treat retinopathy of prematurity, but compared to many years of data on other drugs, the information is still quite sparse, but it appears to be quite consistent.

### 4.1. Effectiveness

In the first studies of retinopathy of prematurity with aflibercept, children were given a dose of 0.025 mL (1 mg), half the recommended adult dose. From the moment of insertion of a small-capacity Picleo syringe, the administered dose is 0.01 mL (0.4 mg) and treatment may be repeated after 6 months. Comparing studies that used both doses can be difficult, so a list of studies was therefore prepared in which the same concentration of aflibercept was used, i.e., 1 mg. Table 2 presents data from studies conducted in several countries—their small number and the small number of patients analyzed are noteworthy.

Characteristic of the described study group is the extremely low birth weight of the treated children (700 ± 240 g), being most similar to the data from the study from Taiwan, in which, however, only five children were treated [6]. Most of the data concern premature babies with a birth weight above 1000 g (low birth weight) [8,11,12]. Children with extremely low birth weight are particularly burdened and at risk of disease reactivation, hence the likely higher incidence of ROP recurrence in our group. Side effects may also be more common in this group due to the immaturity of the nervous and other systems. What is noteworthy is the high percentage of disease recurrence (as much as 45%) in this study, which may be related to the aforementioned low birth weight and, consequently, to the immaturity of the body and a greater number of general burdens in newborns. We observed recurrence between 35 and 40 weeks of postconceptional age, on average, at a PCA of 37.2 weeks. Treatment in this case involved laser photocoagulation of the avascular peripheral retina.

We did not administer anti-VEGF twice, as in studies, e.g., by Salman from 2015 [4], Vural from 2018 [7], and Sukgen from 2019 [8]. The disease recurred in five out of 11 children (10 eyes), while in the cited studies in two, two, and four eyes, the birth weight of the examined children was on average 677 g in our study and 991 g, 1297 g, and 1157.6 g, respectively, in the cited studies. A similar percentage of eyes required laser therapy in the Ekinci study [9] (41.6%), and a higher percentage in the Riazi-esfahani study [10], even though the average birth weight of the children was higher.

All children displayed complete regression of retinopathy of prematurity.

The effectiveness of aflibercept is estimated at approximately 81.9% on average [11]. A large meta-analysis estimated the effectiveness at 80.7% [13]. In those quoted in table studies, it ranged from 86.1% to 100%. Relapses after treatment with this drug are estimated at 13.9% [8] and 28.9% [11], but later stages of ROP are also considered, e.g., 23.2% [12]. The latest reports indicate much more frequent relapses of the disease—on average up to 41.5%—after administering the latest anti-VEGF drugs, especially in the form of A-ROP [11], which requires further observations.

In the Butterfleye study, 86% of children treated with aflibercept (0.4 mg) did not require laser therapy, while 14% required further treatment. In the Firefleye study, 93% of children treated with aflibercept (at a dose of 0.4 mg) did not undergo laser treatment, whereas it was necessary for 7%.

### 4.2. Local Complications

In the study group, after aflibercept treatment, one local complication occurred in the form of lens opacity in one eye, which accounted for 4.5%. In the product characteristics, the incidence of cataracts is estimated at 7.9% (applies to all patients, both children and adults). Sukgen experienced complications such as intraretinal hemorrhage in 10 eyes and subvitreal hemorrhage in three eyes after aflibercept administration [5]. A subcapsular cataract developed in one eye, which was associated with damage to the lens by a needle during injection [13].

In the Butterfleye and Firefleye studies, retinal detachment occurred in five and one children, respectively [3,14].

### 4.3. General Complications

We are aware that anti-VEGF drugs affect the development of the brain, kidneys, heart, and lungs, so children underdoing this treatment require urgent, long-term observation. In his study, Clisal and Sukgen measured blood flow in the CRA, which was normal, and no systemic side effects were observed [14] In the Vural study, it was found that the process of “maturation” of the macula was not delayed due to IVA [7]. In his study, Salman found that visual parameters after the follow-up period were comparable to the results of treatment with other anti-VEGF drugs [4].

Aflibercept may affect neurocognitive functions and cause pulmonary hypertension. In our study, neither pulmonary hypertension nor other general disorders were observed, but further overall development of children will be described in a separate study.

In the Butterfleye study, a child died in one case, but it was not related to treatment.

The study group is small, but this is due to the registration of ranibizumab and the subsequent lack of possibility to choose a drug to treat retinopathy of premature infants. The numbers of operations performed by Salman, Sukgen, Vural, and Huang were not large (15, 15 and 18, respectively, nine children 26, 29 and 36, 17 eyes); only the Butterflyeye and Firefleye studies referred to a large group of patients [3,4,6,7,8,14].

There were no general adverse effects following administration of aflibercept, but administration of this drug requires further monitoring and replacement experiences between centers.

## 5. Summary

The choice of treatment for ROP depends more on the stage of the disease than on the child’s weight. In premature infants, the more immature the child, the more extensive the areas of avascular retina [15]. Many studies have compared the age and weight of premature infants born since the beginning of the 21st century [16,17,18]. Currently, children are born smaller and with a lower birth weight, but according to some authors, this does not affect the severity of ROP [19]. Using anti-VEGF medications can postpone the decision to undergo retinal photocoagulation therapy. Ideally, ROP should not reactivate, but many factors influence this, such as the overall burden of the premature infant. Children with ELBW most often develop ROP in the posterior and middle poles of the eyeball (in zones I and II), as well as A-ROP.

The benefits of using intravitreal medications cannot be underestimated. They offer a quick clinical effect, and their administration is a quick procedure that does not always require general anesthesia, only local anesthesia, in the conditions of a sterile treatment room (even at the patient’s bedside). It is general anesthesia that is often a problem, especially in the smallest premature babies, especially those with a rapidly developing form of ROP and requiring treatment with anti-VEGF preparations. General anesthesia can cause respiratory decompensation in a child, cause dangerous bradycardia, be life-threatening, and prolong their stay in the hospital. However, if the patient requires general anesthesia, it is shallower and shorter than in the case of laser therapy. Laser therapy is effective because it physically destroys the area that stimulates excessive angiogenesis, which leads to the development of ROP. Anti-VEGF drugs target retinal and vitreous EGF, but their duration of action is limited. In the case of the smallest and most preterm infants, we anticipate the need for repeat treatment—i.e., another anti-VEGF drug—as is possible with 0.4 mg aflibercept (FDA recommendations in the US), or laser therapy. Intravitreal drug administration does not require a perfectly wide pupil or a completely transparent lens (e.g., in the case of TVL). Over time, it does not cause myopia and does not affect the field of vision because we do not create scars on the periphery of the retina.

However, there are still too few studies comparing new anti-VEGF drugs. Aflibercept is the least frequently used anti-VEGF drug to treat premature infants. In a study by Tsiropoulos et al. comparing the effects of ROP treatment with various anti-VEGF drugs and laser therapy, 81 reports from 2012 to 2020 were analyzed [20]. Only one of these reports concerned treatment with aflibercept, and the treatment group included 51 children [21]. In a meta-analysis of 30 studies (4686 eyes) comparing the incidence and time of ROP reactivation after anti-VEGF treatment, only five of them involved aflibercept [13]. In these studies, 326 eyes were treated with aflibercept—including 150 eyes in the 2022 FIREFLEYE study (outside Europe) [3], 24 eyes (Ekinci-Turkey) [9], and 24 in the 2021 Riazi-esfahani study (outside Europe) [10].

We can assume that after the drug’s registration in the US, it will also become more widely used in other countries.

These drugs differ from each other in structure, point of action, mechanism of action, and speed and duration of action, with each one being effective in inhibiting the development of retinopathy of prematurity. The local and systemic side effects of aflibercept appear to be the same as those for other intravitreal anti-VEGF drugs. Aflibercept, like any VEGF-binding drug, even when administered intravitreally, can cause systemic side effects. VEGF blockade, regardless of the mechanism, reduces the production of nitric oxide (NO) and prostaglandin I-2 (PG I-2). In adult patients, this is most often associated with hypertension and thrombosis, although these complications are more likely to occur after systemic administration of aflibercept. Following intravitreal administration, patients at particular risk (those with a history of stroke) are especially at risk for systemic complications [22]. There is a potential risk of arterial thromboembolic complications, including stroke and myocardial infarction. Clinical trials have observed a low incidence of thromboembolic events, and no significant differences were found between the aflibercept and control groups [23,24]. Systemic side effects result from the drug entering the bloodstream. In adult patients (in clinical trials, approximately 89% (1616 of 1817 patients) of patients randomized to aflibercept were 65 years of age or older, and approximately 63% (1139/1817) were 75 years of age or older) in a pharmacokinetic study, the maximum plasma concentration of free aflibercept (systemic Cmax) was low, averaging approximately 0.02 micrograms/mL (range 0 to 0.054) within 1 to 3 days after intravitreal injection of 2 mg of the drug, and was undetectable in almost all patients two weeks after administration. Aflibercept administered intravitreally every 4 weeks does not accumulate in plasma. Animal models have shown that the mean maximum concentration of free aflibercept is approximately 50 to 500 times lower than the concentration of aflibercept required to inhibit the biological activity of systemic VEGF by 50%.

In children (in the Butterfleye study), after administration of 0.4 mg of aflibercept to both eyes, serum free aflibercept concentrations were 0.583 mcg/dL on the first day and 0.0406 mcg/dL after 28 days [3]. In the Firefleye study, these concentrations were 0.481 mcg/dL on day 1 and 0.13 mcg/dL on day 28 [25].

In animal models, changes in blood pressure were observed after reaching a concentration of approximately 10 micrograms/mL of free aflibercept in the circulation. Blood pressure returned to normal when the level dropped below approximately 1 microgram/mL. In a study of healthy volunteers, it was observed that after intravitreal administration of 2 mg aflibercept to patients, the mean maximum plasma concentration of free aflibercept was more than 100 times lower than its peak concentration. The concentration required to bind half of systemic VEGF (2.91 micrograms/mL) is therefore unlikely. Therefore, systemic pharmacodynamic effects, such as changes in blood pressure, are unlikely

As with all therapeutic proteins, there is a risk of aflibercept immunogenicity. Antibodies to aflibercept following administration to one or both eyes over a period of 24 to 100 weeks were observed in less than 1% of children, and in adults, in 1 to 3% of patients [25].

Anti-VEGF drugs are becoming the first-line treatment for ROP type I when laser therapy is technically difficult or requires the destruction of a large area of the retina, especially its central part. The most commonly used drugs for years have been bevacizumab, ranibizumab, and aflibercept, but bioequivalent drugs are currently appearing on the market, which require studies on the safety and efficacy of ROP treatment.

Aflibercept appears to be a relatively well-known and safe drug. Of course, it requires long-term follow-up, especially in children. Clinical studies show that the incidence of serious side effects (SAEs) following aflibercept treatment is low. As mentioned, these mainly affect adult patients with other ophthalmological indications for treatment, but also those with other risk factors and older patients.

In the Firefleye-next study, ophthalmological and neurological assessments were performed after 2 years in children treated with aflibercept. The study included 100 children of various races from various countries (21 Asian, two Black, 75 Caucasian, two mixed race). Retinal health, particularly its vascularity and visual function, was assessed. Of the 66 children treated with aflibercept, 62 did not exhibit adverse structural changes, and 61 of 63 did not have ROP. Four children were treated for reactivation of ROP before the age of 1 year. No side effects on development, growth, or neurological status were observed [26]. The Butterleye-next study is currently ongoing and is scheduled to end in November 2026. Ophthalmological and neurological status will be assessed in this study using selected scales [27].

According to authors specializing in the screening and treatment of ROP, it is important to collect local data on the incidence, screening, and treatment methods from around the world [28]. They also emphasize the role and balance between two treatment strategies: laser therapy, used in ROP since the 1990s, and anti-VEGF drugs (used since 2007) [2].

We believe that describing another ethnic group (Caucasian, the first such study from Poland), even concerning a small group of children, may be important in the future.

Intravitreal drugs allow the treatment of the smallest premature infants in whom the rapid development and location of the disease make transpupillary photocoagulation impossible or who are at high risk in the peri- and postoperative period.

All children treated with aflibercept included in this study remain under the care of our center. They were monitored until their retina was fully vascularized or until the vascularization reached the limit of laser therapy. The children’s vision development and refractive error, as well as their overall development, were then checked. Late observations regarding the ophthalmological and general condition of the children will be described in a separate study. The group is small, but most likely the only one in Poland and one of the few in Europe. Due to the interest in the use of aflibercept in treating retinopathy of prematurity, any report on its use to treat ROP and the long-term impact on the ophthalmological and general developmental condition of children is valuable.

## Figures and Tables

**Table 1:** Characteristics of 11 premature infants treated with aflibercept.

Case No.	Sex	BW, g	PCA, Weeks	Stage and Zone		Anty-VEGF/PCA at Treatment, Weeks	Laser/PCA at Treatment, Weeks
				Right Eye	Left Eye		
1.	M	850	24	II 3 (+)	II 3 (+)	33	40
2.	F	800	24	II 3 (+)	II 3 (+)	32	-
3.	F	750	25	A-ROP	A-ROP	33	-
4.	M	540	24	II 3	II 3	33	37
5.	M	850	27	I/II 3 (+)	II 3 (+)	38	-
6.	F	460	23	I/II 3 (+)	I/II 3(+)	35	-
7.	F	500	26	I/II 3 (+)	I/II 3 (+)	33	35
8.	M	700	23	II 3 (+)	II 3 (+)	32	-
9.	M	570	27	A-ROP	A-ROP	32	37
10.	M	940	27	A-ROP	A-ROP	32	37
11.	F	490	23	A-ROP	A-ROP	34	-

**Table 2:** Comparison of ROP treatment studies with aflibercept 1 mg.

Study	Number of Examined Children/Eyes	BW (g)	Regression, Eyes (%)	Recurrence, Type of Treatment (%)
Salman et al. 2015, Egypt [4]	15 (26)	875–1115 (991 ± 266)	25 (96.2)	2 eyes, aflibercept (3.8)
Sukgen et al. 2017, Turkey [5]	15 (29)	1198.62 ± 348.99	29 (100)	0
Huang et al. 2018, Taiwan [6]	5 (9)	476–1160 (764 ± 214.1)	9 (100)	0
Vural et al. 2018, Turkey [7]	28 (36)	640–2290 (1297 ± 540)	34 (94.4)	2 eyes, aflibercept (5.6) late recurrence of ROP in 19.4%—did not require treatment
Sukgen et al. 2019, Turkey [8]	36 (72)	680–1950 (1157.6 ± 298.2)	62 (86.1)	4 eyes aflibercept, 6 eyes LPC (13.9)
Ekcini et al. 2020, Turkey [9]	12 (24)	1095 ± 442	14 (58.4)	10 eyes, LPC (41.6)
Riazi-Esfahanial. et al. Iran, 2021 [10]	12 (24)	730–1975 (1205 ± 383)	10 (41.7)	14 eyes (58.3%)

## Data Availability

The datasets presented in this article are not readily available because [the data are part of an ongoing study]. Requests to access the datasets should be directed to [marbia@poczta.fm].

## Abbreviations

ROP	retinopathy of prematurity
A-ROP	aggressive retinopathy of prematurity
VEGF	vascular endothelial growth factor
VEGFR	vascular endothelial growth factor receptor
AMD	age-related macular degeneration
PFA	patent ductus arteriosus
IVH	intraventricular hemorrhage
PCA	postconceptional age
BW	birth weight
GA	gestational age
IVA	intravitreal aflibercept
CRA	central retinal artery
TVL	tunica vasculosa lentis
